# Pama–Nyungan grandparent systems change with grandchildren, but not cross-cousin terms or social norms

**DOI:** 10.1017/ehs.2020.31

**Published:** 2020-06-05

**Authors:** Catherine Sheard, Claire Bowern, Rikker Dockum, Fiona M. Jordan

**Affiliations:** 1 School of Earth Sciences, University of Bristol, Bristol BS8 1TQ, UK; 2 Department of Anthropology and Archaeology, University of Bristol, Bristol BS8 1UU, UK; 3 Department of Linguistics, Yale University, New Haven. CT 06520, USA

**Keywords:** Pama–Nyungan, phylogenetic comparative methods, kinship, grandparents, cultural evolution

## Abstract

Kinship is a fundamental and universal aspect of the structure of human society. The kinship category of ‘grandparents’ is socially salient, owing to grandparents’ investment in the care of the grandchildren as well as to older generations’ control of wealth and cultural knowledge, but the evolutionary dynamics of grandparent terms has yet to be studied in a phylogenetically explicit context. Here, we present the first phylogenetic comparative study of grandparent terms by investigating 134 languages in Pama–Nyungan, an Australian family of hunter–gatherer languages. We infer that proto-Pama–Nyungan had, with high certainty, four separate terms for grandparents. This state then shifted into either a two-term system that distinguishes the genders of the grandparents or a three-term system that merges the ‘parallel’ grandparents, which could then transition into a different three-term system that merges the ‘cross’ grandparents. We find no support for the co-evolution of these systems with either community marriage organisation or post-marital residence. We find some evidence for the correlation of grandparent and grandchild terms, but no support for the correlation of grandparent and cross-cousin terms, suggesting that grandparents and grandchildren potentially form a single lexical category but that the entire kinship system does not necessarily change synchronously.

## Graphical abstract

**Figure:**
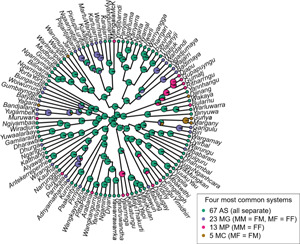

**Media summary:** Across 134 Australian languages, Pama–Nyungan systems for naming grandparents shift with grandkids but not social norms.

## Introduction

Kinship is a basic and universal component of the structure of human society (Keesing, 1975; Parkin, 1997) and was one of the foundation concepts in modern anthropology (Morgan, 1871). Kinship organisation forms the backbone of societal rules for inheritance, marriage, alliance and trade (Chapais, 2014; Opie et al., 2014), which in turn affect individuals’ reproductive fitness (Chagnon et al., 2017) and shape interactions in daily life (Reiss, 1962). Kinship categories are also clearly lexicalised in the world's languages (e.g. Murdock, 1968; Woodward, 1978; Haspelmath et al., 2001). It is often the case that a single kinship word refers to multiple categories of people, such as the English word ‘aunt’ meaning both one's father's sister and one's mother's sister. Although the combinatorial possibilities are very large for categorisations that could exist, in practice only a small fraction of possible kinship systems are found cross-culturally (Kemp & Regier, 2012; Nerlove & Romney, 1967). Indeed, the comparative study of so-called kinship ‘typologies’, or common patterns of classifications of kin, dates to the nineteenth century, although it is only recently that evolutionary anthropologists have returned to the topic (Jordan, 2011), and the extent to which kinship systems change as a single unit remains largely unknown (Godelier, 2012; Godelier et al., 1998). One outstanding question is the degree to which systems of meaning embedded in language – kinship terminologies – have coevolved with, or are predicted by, patterns of normative social behaviour in ethnolinguistic groups. This tension has characterised kinship studies since Kroeber (1909) and Rivers (1914). Some recent scholars have re-visited these questions with richly nuanced ethnographic and linguistic data (see for example studies in McConvell et al., 2013; and Birchall & Jordan, 2019), and here we advocate that an evolutionary cross-cultural approach can help to detect potential relationships between cognition, language and culture.

In the nearly 140 years between Morgan and the re-emergence of cross-cultural kinship study, the discipline of cultural phylogenetics has emerged to examine the evolution of cultural and linguistic traits over time (Atkinson & Gray, 2005; Mace et al., 1994). Although the pseudo-replication caused by related societies’ shared common descent has been noted since the 1880s (Tylor, 1889), it was not until the late twentieth century that statistical methods were invented to study the evolution of biological traits along phylogenetic trees (Harvey & Pagel, 1991), and it was a few years later that these techniques were applied to human language and culture (Blute & Jordan, 2018; Gray et al., 2007; Mace & Holden, 2005; Mace et al., 1994). Since then, the study of cultural evolution using phylogenetic comparative methods has been utilised on a wide variety of cultural traits, from the evolution of socio-political hierarchies (Sheehan et al., 2018) to the spread of folktales (da Silva & Tehrani, 2016). A handful of these studies have focused on kinship systems. Jordan (2011), for example, investigated Austronesian sibling terminologies to show that the relative-age distinction (i.e. older vs. younger sibling) predated distinctions in relative gender (i.e. same-gender sibling vs. opposite-gender sibling). Bantu kinship typologies, which are based on Murdock's (1949) discrete classifications of kinship systems using the number and types of words for cousins, were shown by Guillon and Mace (2016) not to correlate with descent or residence rules, but these rules do correlate with sex biases within the systems (Opie et al., 2014). A recent study found that for a mostly global sample of 936 languages, these Murdock cross-cousin terminologies correlate with community marriage and descent rules (Rácz et al., 2019). To date, however, there has been no cross-cultural phylogenetic study of grandparent terminologies.

Grandparents are an important social category. Grandparents, particularly grandmothers, are a significant source of ‘alloparenting’, both throughout our evolutionary history (Hrdy, 2011) and in the modern day (Normile, 2017). Researchers have demonstrated a cross-cultural matrilateral bias in the investment of grandparents; across many (but not all) societies, the maternal grandmother, whose genetic relation to the child is certain, provides the most extra-parental help in raising the child (Pashos, 2017; Perry & Daly, 2017) and can significantly improve grandchild survival and nutritional status (Sear & Mace, 2008; Sear et al., 2000). The human post-reproductive lifespan, a rarity among mammals, has been posited as an adaptation that specifically allowed grandmothers to help raise their grandchildren in what is known as the ‘grandmother hypothesis’ (Hawkes et al., 1998; K. Hill & Hurtado, 1991). Grandparents play a variety of significant cultural and social roles within a society, as older individuals tend to control a considerable amount of wealth and skills (Møllegaard & Jæger, 2015). Moreover, specific terms that record further details about the social category of the grandparent – for example, that a person is a grandmother rather than a grandfather or a paternal grandmother rather than maternal one – can aid in the categorisation of one's kin by socially salient categories, such as keeping track of moieties, noting lines of inheritance, or working out preferred marriage partners (Chapais, 2014).

Across the world's languages, classification systems for grandparents vary. Many dialects of English have just two terms for grandparents, ‘grandmother’ and ‘grandfather’, which distinguish gender but not lineage (i.e. paternal vs. maternal grandparents); in contrast the Australian language Alyawarr has four unique terms for each role (Yallop, 1977), while the Hawaiian words *tutu* or *kuku* can refer to any grandparent. As with the more famous kinship systems based on cousin terms (Goody, 1970; Guillon & Mace, 2016; Rácz et al., 2019), cross-cultural variation in grandparent systems is thought to vary with aspects of social structure, such as community marriage organisation (Dole, 1969; McConvell & Dousset, 2012; Parkin, 2012) and post-marital residence (McConvell, 2012), although the extent to which grandparent terms change in concert with other categories of kinship is unknown (Dzibel & Dziebel, 2007; Read, 2013). Ethnolinguistic groups do not, however, represent independent samples. Vertical transmission between historically related societies often shapes extant patterns, necessitating phylogenetic models in the study of cross-cultural variation (Gray et al., 2007; Mace et al., 1994). Horizontal transmission, or borrowing between cultures in contact, can also determine traits’ evolutionary trajectories (Currie et al., 2010; Nunn et al., 2006). A study of the relationship between social practices and grandparent systems (or indeed any kinship classification) would therefore need to account for the potential of both historical and spatial autocorrelation. As a well-resolved global language phylogeny is currently lacking, and given the current body of evidence suggesting the substantial role local processes can play in cultural macroevolution families (Dunn et al., 2011; Fortunato & Jordan, 2010; Moravec et al., 2018; Surowiec et al., 2019), this limits a study of the variation in grandparental systems to the scale of a language family.

Pama–Nyungan is the world's largest hunter–gather language family, comprising over 300 languages traditionally spoken across a region encompassing over 90% of the Australian continent (Bowern & Atkinson, 2012). Although Aboriginal Australians have inhabited Australia for over 50,000 years (Malaspinas et al., 2016), Pama–Nyungan probably originated between 4,500 and 7,000 years ago in the Gulf Plains region and appears to have quickly spread together with cultural innovations such as agricultural intensifications and rock art (Bouckaert et al., 2018). During this expansion, there is ambiguous evidence for genetic replacement, suggesting that these languages spread through assimilation rather than comprehensive takeovers (Bouckaert et al., 2018). The approximately 100 non-Pama–Nyungan languages found in Australia are all restricted to the north coast; beyond divisions into 27 linguistic families, the phylogenetic relationships among these groups remain opaque (McConvell & Bowern, 2011). Within the domain of kinship specifically, Australian societies are notable for social categories such as moieties, skins and sections (Lacrampe et al., 2018; Scheffler, 1978). Generational moieties, such as those found in the Western Desert languages, classify grandparents and grandchildren in the same moiety (White, 1981); in these languages, grandparent and grandchild terms are frequently colexified. Some Pama–Nyungan languages, such as Yidiny and Dyirbal, also have kinship avoidance registers, where sons-in-law and mothers-in-law observe strict behavioural and linguistic taboos in one another's presence, such as using a separate avoidance vocabulary (Dixon, 1990). Linguistic exogamy is also common in Australia (Clendon, 2006; J. H. Hill, 1978), which has been demonstrated to affect borrowing rates generally (Bowern et al., 2011) and kinship term borrowing specifically (Bowern, 2010; Haspelmath, 2008).

The large sample size of Pama–Nyungan languages within a single well-resolved phylogeny (Bouckaert et al., 2018; Bowern & Atkinson, 2012), coupled with a continent-level radiation of languages, permits the use of phylogenetic comparative methods to investigate the co-evolution of language and culture across space and time. By focusing explicitly on a group of hunter–gatherer languages, we are able to model the dynamics of language change without the potential confounding effects of the many cultural and political changes that accompanied domestication and agricultural transitions (Creanza et al., 2017). Farming practices date to approximately 11,000 years ago (Bramanti et al., 2009), whereas the Upper Palaeolithic transition, characterised by the sudden appearance of technological and cultural complexity in early modern humans, was approximately 45,000 years ago (Powell et al., 2009); comparative studies of wholly agricultural populations therefore may not be representative of the entirety of human evolution (Bowern et al., 2011). Moreover, even if there is no difference in the evolutionary dynamics of hunter–gatherer and of agriculturalist kinship systems, the unique biogeographic and anthropological history of each region is expected to separately shape different language families (Dunn et al., 2011; Fortunato & Jordan, 2010; Moravec et al., 2018). Phylogenetic comparative analyses of aspects of kinship systems have been performed on Indo-European (Fortunato & Jordan, 2010), Bantu (Guillon & Mace, 2016; Opie et al., 2014) and Austronesian languages (Fortunato & Jordan, 2010; Jordan, 2011), as well as a small number of cross-family studies (Boden et al., 2014; Moravec et al., 2018; Rácz et al., 2019; Walker & Bailey, 2014), but Australian studies have been largely absent from this literature.

Here we use Bayesian phylogenetic comparative analyses to reconstruct the sequence of changes in Pama–Nyungan grandparent term systems and to test the role of social structure in shaping these changes. Specifically, we evaluate the effects of phylogenetic signal (vertical transmission), spatial signal (horizontal transmission), community marriage organisation (specifically, the effect of linguistic exogamy) and post-marital residence. Finally, because variation in kinship terminologies is often collapsed into a small number of typologies (Dzibel & Dziebel, 2007; Fox, 2008; Murdock, 1949; Read, 2013) – typically based on cousin terminology, and often applied without validating that these classifications are meaningful in the sample under study – we test for kinship system synchrony within our sample. We thus compare changes in grandparent systems with (1) changes in grandchild term systems and (2) changes in terms for cross-cousins.

## Methods

### Data collection

Terms for grandparents were obtained from the Australian linguistics database CHIRILA (Bowern, 2016) for 134 Pama–Nyungan languages. Here ‘grandparents’ are defined as the linguistic terms for a speaker's mother's mother (MM), mother's father (MF), father's mother (FM) and father's father (FF); definitions of common abbreviations and terms can be found in Table 1. These terms were then categorised based on which grandparent terms, if any, were merged; in Pama–Nyungan, the relevant distinctions are between the two parallel grandparents (MM and FF), the two cross grandparents (MF and FM), the two female grandparents (MM and FM) and the two male grandparents (FF and MF). These scores formed the basis of a discrete post-hoc categorisation into ‘systems’ (Table 2).

**Table 1.** Glossary of kinship terms and abbreviations. These terms are not exhaustive and do not encompass the diversity of human experience (e.g. step-grandparents, same-gender marriages, non-binary genders, differentiation between sex and gender). By convention, kinship terms are typically abbreviated such that WXY is interpreted as W's X's Y. M = mother, F = father, D = daughter, S = son, Z = sister and B = brother

MM	Speaker's mother's mother; maternal grandmother
MF	Speaker's mother's father; maternal grandfather
FM	Speaker's father's mother; paternal grandmother
FF	Speaker's father's father; paternal grandfather
Cross grandparents	Opposite-gender parents of speaker's parents; MF and FM
Parallel grandparents	Same-gender parents of speaker's parents; MM and FF
Community marriage organisation	General community norms toward marriage within or between groups. Here we focus on specifically *linguistic* exogamy, or marriage outside of an ethnolinguistic group. Endogamy: marriage within the (here, linguistic) group. Agamy: no tendency to either endogamy or exogamy. See Kirby et al. (2016) and Murdock (1967) for more information
Post-marital residence	The prevailing pattern of residence of a couple after marriage. Patrilocal: newly married couple typically resides near the husband's family. Matrilocal: newly married couple typically resides near the wife's family. See Kirby et al. (2016), Moravec et al. (2018) or Murdock (1967) for more information

For 58 of these languages, we were able to obtain further data on the terms for ‘grandchild’ (56 languages) and/or ‘cross-cousin’ (51 languages). Most of this data came from the Australian language database CHIRILA (Bowern 2016) and the social organisation and kinship database Austkin (Dousset et al. 2010); full citations for each language are provided in the supplementary material.

As with grandparents, there are four core grandchild categories: daughter's daughter (DD), daughter's son (DS), son's daughter (SD) and son's son (SS). In Pama–Nyungan languages, it is common for grandchild and grandparent terms to be colexified; we thus noted if any grandchild term was the same as any grandparent term. Because grandchildren terms tended to be complicated, we devised a post-hoc coding scheme based on four binary variables: (1) whether any of the grandchild terms was a generic (a term for which DD = DS = SD = SS); (2) whether any of the terms were merged by gender of the referent (DD = SD and/or SS = DS); (3) whether any of the terms were merged by the parent of the referent (DD = DS and/or SS = SD); and (4) whether each grandchild could be assigned a unique term.

Cross-cousin terms were coded according to a similar set of four criteria: (1) whether there was a generic term that could be applied to all four cross-cousins; (2) whether any terms were merged by gender of the referent (MBD = FZD and/or MBS = FZS); (3) whether any terms were merged by the parent of the referent (MBD = MBS and/or FZD = FZS); and (4) whether each cousin could be assigned a unique term.

### Ancestral dynamics of grandparents

All phylogenetic analyses were performed on a distribution of 100 trees from Bouckaert et al. (2018), which contained tips for 110 of the 134 languages surveyed. As many comparative analyses require ultrametric trees, the tips of all recently extinct languages were extended to the present day. To assess the phylogenetic signal of the grandparent systems, we used a *λ*-transformation using the command ‘fitDiscrete’ in the R package *geiger* (Harmon et al., 2008). The ancestral grandparent system was calculated for each tree in a maximum likelihood framework using the command ‘ace’ in the R package *ape* (Paradis et al., 2004); the median values across the 100 trees are reported here, and the results computed on a consensus tree from this distribution are shown in Figure 2. The Bayesian analysis to reconstruct the dynamics of grandparent system transitions also produced an estimation of the root ancestral state (see below); we report these additional results for completeness. Further details on these analyses can be found in the supplementary material.

To assess the potential for spatial signal – which could be a result of horizontal transmission and/or an indication that grandparent systems are partially driven by local environmental variables – we obtained latitude and longitude coordinates of point estimate locations for 130 of the 134 languages from Glottolog (Hammarström et al., 2018), with coordinates from the remaining four languages provided by author CB. We then performed a Mantel test using the command ‘mantel.rtest’ from the R package *ade4* (Dray & Dufour, 2007), which tests for correlation between two matrices, with 9,999 permutations. The distance matrix was taken as straight-line distances between each language's point estimate; the grandparent system ‘distance’ was set to be 0 if languages shared a system and 1 otherwise.

To assess the internal dynamics of grandparent systems, we used the command ‘Multistate’ in BayesTraits (Pagel et al., 2004), which calculates the transition rate between systems. To minimise the number of parameters to be estimated with this relatively small sample size, we limited our analysis to the four most common grandparent systems – (1) four separate terms, (2) merging the genders, (3) merging the parallel grandparents and (4) merging the cross grandparents (see Table 2) – and employed a Markov chain Monte Carlo (MCMC) reversible jump process, which allows parameters to be set to 0. All priors were set to be drawn from an exponential distribution with a mean of 10, and for each tree the chain was run for 1,010,000 iterations with a burn-in of 10,000 and a sampling rate of 1,000. We report the median values of each parameter across all tree topologies in the distribution. This analysis also provides a Bayesian framework in which to estimate the root ancestral state, in a model allowing transitions between the four most common systems to vary asymmetrically; these results are presented alongside the maximum-likelihood ancestral state estimates (see above).

**Table 2.** Pama–Nyungan grandparent systems. Languages were first coded by whether they had the same words for female grandparents (MM = FM), male grandparents (MF = FF), parallel grandparents (MM = FF) and cross grandparents (MF = FM), and then classified into discrete systems

Description	Abbreviation used in-text	Kinship equivalences	Number of languages	Percentage of languages	Example language
All separate terms	AS	none	86	64%	Alyawarr
Merge by gender	MG	MM = FM, FF = MF	23	17%	Pitjantjatjara
Merge ‘parallel’ grandparents	MP	MM = FF	13	10%	Djapu
Merge ‘cross’ grandparents	MC	MF = FM	6	4%	Yagara
Merge cross and parallel	—	MF = FM, MM = FF	4	3%	Kuku Yalanji
Merge by gender of parent	—	MM = MF, FM = FF	1	<1%	Nyawaygi
Merge grandmothers	—	MM = FM	1	<1%	Wemba Wemba

### Co-evolution between grandparent system and cultural norms

We obtained information on the community marriage organisation for 29 societies from D-PLACE (Kirby et al., 2016), which for Australian data is based on Binford (2001)'s cross-cultural survey of hunter–gatherer societies. D-PLACE scores ‘community marriage organisation’ using five categories, four of which were found in our sample. We collapsed this to three categories: exogamous, agamous and endogamous (D-PLACE ‘endogamous deemed’ and ‘endogamous segmented’). Data for a further 46 languages were obtained from the ethnographic literature; a list of sources and scores is available in the supplementary material. We also obtained information on community post-marital residence norms for 50 languages from Moravec et al. (2018). All languages in our sample are either patrilocal (newly married couples typically reside near the husband's family) or matrilocal (newly marriage couples typically reside near the wife's family).

To assess the relationship between community marriage rules and grandparent system, we ran binary Bayesian phylogenetic mixed models (BBPMM) using the R package *MCMCglmm* (Hadfield, 2010) across a sample of 100 trees; full details of model parameters can be found in the supplementary material.

To ensure model convergence when testing the relationship between grandparent systems and community marriage rules, we restricted our sample to only the two most common grandparent systems (four separate terms and merging by gender). Because we were specifically interested in the effect of linguistic exogamy on grandparental systems, we also merged endogamy and agamy into a single category for the analyses reported here. (A set of analyses with agamy and exogamy instead merged is reported in the supplementary material.) Owing to the long history of presenting cross-cultural data without a phylogenetic correction (for recent examples, see Rácz et al., 2019; Schulz et al., 2019; or Whitehouse et al. 2019), we also assessed the relationship between grandparent terminologies and marriage systems in a non-phylogenetic framework, the results of which we report in the supplementary material.

To further investigate the precise drivers behind this apparent relationship between grandparent systems and social norms, and in an attempt to increase our sample size, we also tested the relationship between both community marriage organisation and post-marital residence with each of the four main features of Pama–Nyungan grandparent systems (and the basis of our original coding scheme): merging the female grandparents (MM = FM), merging the male grandparents (FF = MF), merging the cross-grandparents (MF = FM) and merging the parallel-grandparents (MM = FF).

### Co-evolution with grandchild and cross-cousin systems

Finally, we assessed the internal synchronicity of the Pama–Nyungan kinship system by evaluating the co-evolution of grandparent systems with two other other kinship categories, grandchild and cross-cousin systems. We first considered whether polysemy between grandparent and grandchild terms is related to grandparent systems; the phylogenetic results are presented here, with non-phylogenetic results presented in the supplementary material. We then ran BBPMMs comparing grandparent systems and each of three traits of grandchild and cross-cousin systems: (a) whether a single generic word can be applied to all members of the category; (b) whether the system merges terms by gender of the referent; and (c) whether the system merges terms by the parent of the referent.

## Results

### Grandparent systems: an overview

The most common system, found in 86 languages, had four separate terms for all four kinship roles (here abbreviated AS). The second-most common system, found in 23 languages, merged the gender of the referent (MM = FM and FF = MF, as in English, abbreviated MG). The next most common system, found in 13 languages, had a single term for the so-called ‘parallel’ grandparent, the parent's same-gender parent (MM = FF, abbreviated MP). The four remaining systems are described in Table 2.

**Figure 1.**
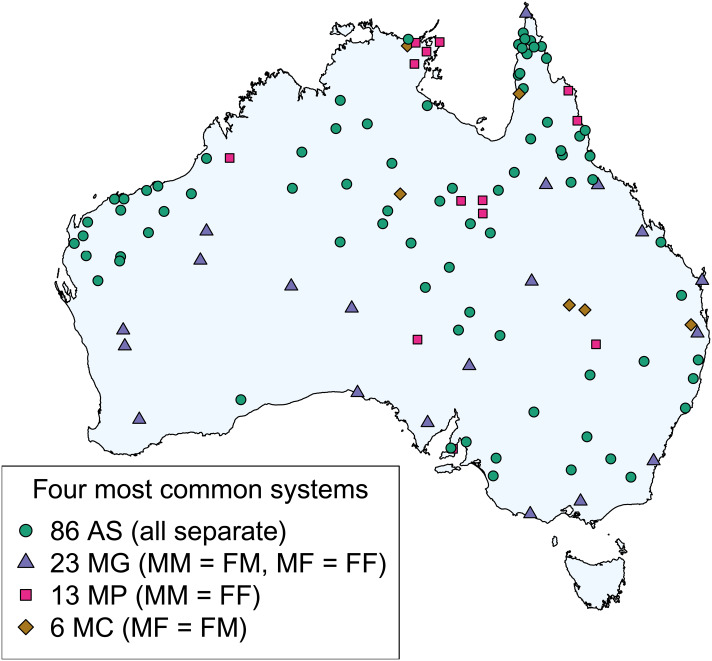
Distribution of Pama–Nyungan grandparent systems across space. Each system is plotted at the society's centroid. The three least common systems (six languages) are omitted.

Grandparent systems showed a strong phylogenetic signal (AIC*_λ_* = 286.7, AIC*_λ=1_* = 286.7, AIC*_λ=0_* = 348.8) but showed no geographic signal (Mantel test, *P* = 0.417) (Figure 1); geographic distance was thus disregarded as a potential confounding variable in all further analyses.

### Ancestral dynamics of grandparents

**Figure 2.**
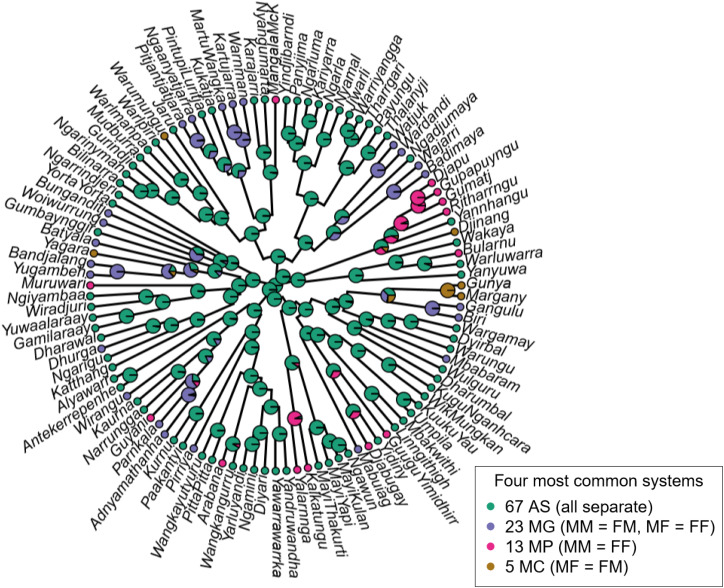
Distribution of Pama–Nyungan grandparent systems across time. The least common systems (two languages) are omitted. For the purposes of display, the topology shown is a consensus tree from the posterior distribution in Bouckaert et al. (2018), and ancestral states assume equal transition rates between all states; the results presented in-text are the median values across the 100 tree topologies.

With very high probability, the ancestral grandparent system of all Pama–Nyungan languages was AS, four separate terms (*P* = 0.9996 assuming equal rates, *P* = 0.9991 assuming symmetric rates) (Figure 2). This remained true even when rare systems were excluded from the analysis (*P* = 0.9993 assuming equal rates; *P* = 0.9990 assuming symmetric rates; or *P* = 0.9989 allowing asymmetric rates in a Bayesian framework). For the dynamics reconstructed on the consensus tree, there were at least nine independent instances of merging grandparents by gender (MG), eight independent instances of merging parallel grandparents (MP) and four independent instances of merging cross grandparents (MC). Although a large number of independent cultural innovations can be indicative of high rates of horizontal transmission, given the lack of geographic signal and the high phylogenetic fidelity, these transitions are more likely to be the products of a flexible trait.

**Figure 3.**
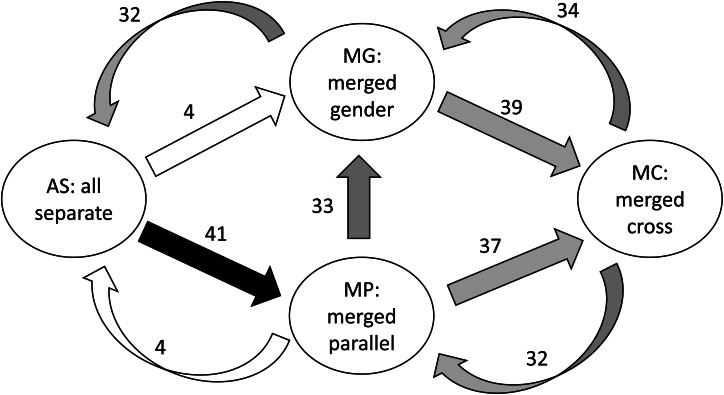
Evolutionary transitions between grandparental systems. The ancestral state has four separate terms for the four grandparents (AS); this state transitions to either a three-term system with merged parallel grandparents (MM = FF, MP), or with an order of magnitude smaller probability, to a two-term system with merged genders (MM = FM and MF = FF, MG). These secondary states could then subsequently transition to a three-term state with the cross grandparents merged (FM = MF, MC). Arrow colour indicates the modelled transition rate, *q*, over an infinitely small time period; see Pagel (1994) for more details. An absent arrow (e.g. from MS to MP or between AS and MC) indicates that this transition rate was estimated as 0.

Among the four most common grandparent systems, the ‘Multistate’ analysis reconstructed a high rate of transition between the ancestral four-term AS system and a three-term MP system (MM = FF) (median instantaneous transition rate q = 41), with some transitions also reconstructed from the four-term system to a two-term MS system (MM = FM and MF = FF) (*q* = 4) (Figure 3). Both of these states would then with high probability undergo a subsequent transition to the three-term MC system (MF = FM) (*q*_MP→MC_ = 37 and *q*_MS→MC_ = 39), with additional transitions from MP to MS (*q* = 33). We observed on average no transitions between the tertiary state (MC) and the primary state (AS) (*q*_AS→MC_ = 0, 95% credible interval 0–27; *q*_MC→AS_ = 0, 95% credible interval 0–106), nor any transitions from secondary state MS to secondary state MP (*q* = 0, 95% credible interval 0–58). Although it might be reasonable to assume that some of these shifts are the result of contact, particularly in multilingual and/or exogamous communities where parents and grandparents may be from different language backgrounds, we found no overall evidence of spatial structuring within Pama–Nyungan languages.

### Co-evolution with community marriage organisation

**Figure 4.**
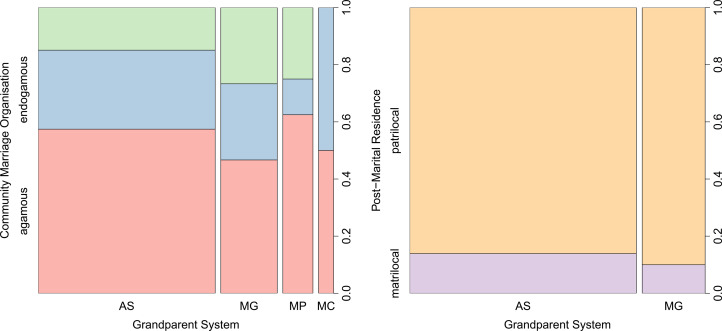
The distribution of Pama–Nyungan grandparent systems by social norms. AS, all separate; MG, merged genders (MM = FM and MF = FF); MP, merged parallel (MM = FF); and MC, merged cross (FM = MF). The differences shown are not statistically significant.

Of the 75 languages for which we were able to obtain data on community marriage rules, one (Warumungu) had a unique grandparent system and was therefore omitted, leaving a total of 74 languages across four systems. We expected to find that societies with more potential resources for term innovations – i.e. those with linguistic exogamy, vs. linguistic endogamy – would have a greater number of terms. We found, however, no effect of community marriage organisation on grandparental systems (pMCMC = 0.928, Figure 4). Similarly, we found no evidence of a relationship between linguistic exogamy and any individual component of grandparental systems across all 75 languages, namely merging the grandmothers (MM = FM, pMCMC = 0.802), merging the grandfathers (MF = FF, pMCMC = 0.836), merging the cross grandparents (MF = FM, pMCMC = 0.162) or merging the parallel grandparents (MM = FM, pMCMC = 0.918).

### Co-evolution with post-marital residence

Of the 50 languages for which we were able to obtain data on post-marital residence norms, four had grandparent systems found in only one or two languages and were thus omitted, leaving a sample of 46. Owing to the differential resource allocation in grandchildren between maternal and paternal grandparents (Pashos, 2017), as well as long-standing beliefs that post-marital residence shapes kinship systems more generally (Murdock, 1949), we expected to see differences in the types of grandparental systems employed by matrilocal and patrilocal societies. We observed, however, no such effect (Figure 4, pMCMC = 0.730).

As with community marriage organisation, we also found no evidence that post-marital residence correlates with any particular aspect of grandparent systems across all 50 languages, namely merging the grandmothers (MM = FM, pMCMC = 0.998), merging the grandfathers (MF = FF, pMCMC = 0.990), merging the cross grandparents (MF = FM, pMCMC = 0.600) or merging the parallel grandparents (MM = FM, pMCMC = 0.496).

### Co-evolution with grandchild and cross-cousin systems

Finally, to test how well Pama–Nyungan grandparent terms fit into the concept of ‘kinship’ as a single synchronous typology, we evaluated the relationship between grandparent systems and grandchildren or cross-cousin terms. Although grandchild and grandparent systems may appear to be correlated, these relationships largely do not survive phylogenetic correction. For example, of the 56 languages for which we were able to obtain grandchild data, 43 languages (77%) have a word that can be applied to both a grandparent and a grandchild category. These grandparent–grandchild reciprocals are common in systems that merge the gender of the grandparent (100%) and in systems with separate terms for each grandparent (85%); it is rarer in systems that merge the parallel (50%) or cross (40%) grandparents. This association between grandparent systems and grandparent–grandchild polysemy, however, was indistinguishable from random variation within a phylogenetic framework (pMCMC = 0.736).

Similarly, MG languages (MM = FM and FF = MF) appear likely to merge the grandchildren terms by the gender of the referent (SS = DS and/or DD = SD) and less likely to merge the grandchildren terms by identity of the parent (SS = SD and/or DD = DS), but these relationships did not hold after phylogenetic correction (pMCMC = 0.408 and pMCMC = 0.288 respectively). MP languages (MM = FF), however, were indeed most likely to have a generic word for ‘grandchild’, independent of shared cultural history (pMCMC < 0.001).

There was no apparent relationship between the four most frequent grandparental systems and whether the cross-cousin systems merge by gender (Fisher's exact test, *P* = 0.672), merge by lineage (Fisher's exact test, *P* = 0.140), or have four separate terms (Fisher's exact test, *P* = 0.704). A generic cousin term was common in AS (79%) or MG (100%) systems and rare in MC (50%) or MP (38%) systems (Fisher's exact test, *P* = 0.022), but this difference did not survive phylogenetic correction (pMCMC = 0.220).

## Discussion

Here we have shown that, contrary to previous expectations, shifts in Pama–Nyungan grandparent systems do not correspond to changes in community marriage organisation or social norms. Furthermore, although some aspects of grandparental systems seem to change in concert with shifts in grandchild and cross-cousin terms, many of these patterns do not survive phylogenetic correction. Thus, we find no strong evidence of a single set of kinship typologies within Pama–Nyungan, especially one such as those based on cousin terminologies in the sense of Murdock (1949).

We find 10 grandparent systems, out of the 15 combinatorial possibilities, across 134 languages, with most languages falling into a single category (four separate terms, 61%). Given the high phylogenetic signal in the data, some of this under-dispersion could be due to phylogenetic conservatism; grandparent systems could be slow to change. Alternatively, although we find no correlation between grandparent system and the two social variables tested here (community marriage organisation and post-marital residence), grandparent systems could be adaptively flexible to other social, cultural or cognitive drivers. Furthermore, the fact that we find no evidence of spatial correlation could suggest that grandparental systems are not strongly shaped by any drivers that themselves have strong spatial signals (such as an environmental variable) or that kinship systems are not horizontally diffused to nearby populations at any meaningful rate. It is unclear, however, how much these patterns may translate to other groups. For example, an analysis of Khoisan sibling terminology found much less evidence of phylogenetic conservatism within the kinship system (Boden et al., 2014), while the strong support for reconstructed states for most nodes in an analysis of Austronesian sibling terms suggests a strong phylogenetic signature (Jordan, 2011), indicating that regional-level processes may be overriding any universal rules regarding kinship system change. A potential cause of this may be the imprecision of straight-line distance as a proxy for inter-group contact, given the roles geographical features (such as navigable waterways or impassable mountains) or cultural practices can play in facilitating horizontal transmission (Bowern, 2013). Within the Australian context specifically, we here only study Pama–Nyungan languages and thus cannot rule out borrowing from non-Pama–Nyungan languages, particularly in Arnhem Land (Bouckaert et al., 2018; Bowern & Atkinson, 2012; Heath, 1978). It is also important to emphasise the distinction between changes in overall systems (the subject of this study) and changes in terms (upon which we do not remark), as preliminary analyses of Pama–Nyungan sibling terms indicate that lexical items themselves change more frequently than systems (Bowern, 2014).

Furthermore, we find that grandparent systems do not change randomly. Among the four most common systems, we do not observe transitions between all states; instead, the ancestral state of four separate terms can shift to either merged parallel grandparents (MM = FF) or, more rarely, to merged genders (MM = FM and MF = FF), either of which secondary states can subsequently shift to merged cross grandparents (MF = FM). Intriguingly, these shifts appear to occur without evidence of a stable, long-term intermediate stage (for example, from merged parallel to merged cross without either a system where MM = FF and MF = FM or by returning back to four separate terms). A similar phenomenon can be observed in examining the history of the terms themselves. For example, many Pama–Nyungan languages have a term *kami that means MM, while other languages have this term as FM, with no strong evidence of an intermediate stage where *kami means both MM and FM (McConvell, 2013).

Although kinship systems are generally approached from anthropological or linguistic perspectives, they also present a combinatorial puzzle, with important implications for the study of kinship typologies. Classifications of family members can be abstractly conceptualised as partitions of a set, meaning that the number of possible systems for a family of *n* roles corresponds to the *n*th Bell number. Thus, four grandparents, with no mechanism for distinguishing speaker gender, have 15 possible classification systems; while a system with 16 roles (say, cousins that distinguish lineage, referent gender, and speaker gender) has 10,480,142,147 possible classifications. Obviously, not all possible systems are found in the world's languages; if nothing else, there are only approximately 7,000 languages spoken today (Anderson, 2010). Some of this variation is thought to be structured by basic cognitive constraints; Kemp and Regier (2012), for example, surveyed 487 world languages and found 410 different kinship systems that nearly all adhered to a single-dimensional trade-off between simplicity and informativeness. Other variation is thought to be shaped by social structure. For example, Rácz et al. (2019) found that Murdock's cousin typologies correlate with a society's rules for marriage and descent in a sample of 936 languages (none Australian). Within the Australian context, so-called Omaha skewing, or merging terms across generations within a system with separate words for patrilineal cross-cousins, is thought to be related to exogamy to avoid demographic collapse in small population groups (McConvell, 2012; McConvell & Dousset, 2012). On the other hand, Guillon and Mace (2016) find no relationship between Bantu cousin systems and either descent or residence norms, suggesting that not all language–culture coevolution hypotheses with microevolutionary support will hold true at the macroevolutionary level.

Many studies in cross-cultural human kinship attempt to classify the vast variation into a small number of socially meaningful typologies (Dzibel & Dziebel, 2007; Fox, 2008; Morgan, 1871; Murdock, 1949; Read, 2013). Our results suggest that grandparent and cross-cousin systems are uncorrelated, and thus that a Murdock-style classification based only on cross-cousins will obscure variation in other aspects of kinship. Furthermore, many of our correlations between grandparental systems and grandchild systems are only statistically significant when the autocorrelation owing to shared cultural history is ignored (see supplementary material), indicating that observations of kin term synchrony uncorrected for phylogeny may be invalid. Studies of Australian kinship have often discarded the globally based six-category Murdock system in favour of systems that are more meaningful for the continent (McConvell & Hendery, 2017); by studying systematic variation at the level of the term within a phylogenetic context, we are able to go even further and suggest that kin term synchrony, and thus stable kinship typologies, may be rarer than previously thought.

By focusing on a large expansion of hunter–gatherer languages, we are able to complement previous phylogenetic studies of kinship systems in agricultural societies and provide additional data in service of testing regularities in cultural evolution. Humans lived as hunter–gathers for the vast majority of our species’ history, and hunter–gatherer kinship is thought to differ from that of agriculturalists in terms of residence patterns (K. R. Hill et al., 2011), inbreeding rates (Walker & Bailey, 2014) and the speed at which systems can change (Opie et al., 2014). Hunter–gather languages are also generally assumed to have a high level of borrowing (Dixon, 1998; Nettle, 1999), although recent work has demonstrated that loan rates in hunter–gatherer languages from Australia, Amazonia and western North America are broadly comparable with, albeit more variable than, those in neighbouring agricultural languages (Bowern et al., 2011). We find no evidence of spatial autocorrelation in our data, and thus no particular evidence of borrowing. Instead, we find a strong phylogenetic signal and high phylogenetic conservatism, indicating that grandparental systems are largely inherited from previous generations and neither change flexibly nor are commonly borrowed.

In summary, we found no evidence that Pama–Nyungan grandparent systems are related to post-marital residence or community marriage organisation. Most languages in our survey had a system with four separate terms, which occasionally shifted to mergers along either referent gender or the parallel grandparents, with a few languages subsequently shifting to a cross merger. We found no evidence that these shifts in grandparent terms relate to shifts in systems for classifying cross-cousins, although we do find moderate evidence linking grandparental and grandchild terms, indicating that care is needed in the assumption of a small number of kinship typologies, particularly in studies lacking phylogenetic correction.

## Data Availability

The data behind this paper can be found at https://doi.org/10.5281/zenodo.3832215. Full data sources can be found in the supplementary material.

## Supplementary material

For supplementary material accompanying this paper visit http://dx.doi.org/10.1017/ehs.2020.31. click here to view supplementary material
