# Pama–Nyungan grandparent systems change with grandchildren, but not cross-cousin terms or social norms

**DOI:** 10.1017/ehs.2021.43

**Published:** 2021-09-08

**Authors:** Catherine Sheard, Claire Bowern, Rikker Dockum, Fiona M. Jordan

The authors apologise that upon publication the link provided in the Data availability statement was incorrect.

The correct link to find the deposited data is doi.org/10.5281/zenodo.3906666

## References

[ref1] Sheard, C., Bowern, C., Dockum, R., & Jordan, F. M. (2020). Pama–Nyungan grandparent systems change with grandchildren, but not cross-cousin terms or social norms. Evolutionary Human Sciences, 2, E30; doi: 10.1017/ehs.2020.31 35663513 PMC7612801

