# Supplementary material for: Pama–Nyungan grandparent systems change with grandchildren, but not cross-cousin terms or social norms
Source: Evol Hum Sci. 2020 Jun 5;2:e30. doi: 10.1017/ehs.2020.31 (PMC7612801; doi:10.1017/ehs.2020.31)
Supplement: Supplementary file 1 [file S2513843X20000316sup001.pdf]

Supplemental information for: *Pama-Nyungan grandparent systems change with grandchildren, but not cross-cousin terms or social norms*

Catherine Sheard, Claire Bowern, Rikker Dockum, and Fiona M. Jordan

- Further details on phylogenetic methods
- Non-phylogenetic models
- Correlations between grandparental systems and community marriage organisation
- Data sources
- Supplemental references

Further details on phylogenetic methods

Tests of phylogenetic signal

A λ-transformation scales the branches of the tree by a factor, λ, that assesses how well the observed trait distribution matches the expected trait distribution for that tree under Brownian motion. A value of λ=1 indicates a perfect phylogenetic signal and returns an output tree identical to the input; a value of λ=0 indicates no observed phylogenetic signal and returns a star-like phylogeny where the only internal branches are between the tips and the ancestral state, a single unresolved polytomy. To determine the significance of the λ-transformed grandparent tree, we compared the log likelihood of the λ-transformed original tree to models where we forced λ=0 and λ=1 using a likelihood ratio test approximated by a χ^2-distribution with a single degree of freedom.

Ancestral state reconstruction

Ancestral states were calculated on both the full dataset and on a dataset restricted to just the four most common systems, each under two sets of assumptions: (1) equal transitions rates among states, and (2) symmetric transition rates among states (so a transition from state 1 to state 2 is equally probable as a transition from state 2 to state 1, but that probability need not be related to any transitions to state 3). The 'ace' command does allow a third mode, in which all transitions rates are allowed to vary; our sample size, however, was too small to accurately compute so many independent transition rates (12 separate rates on the restricted dataset, or 56 on the full dataset). This command also returns the ancestral states for each internal node; the phylogeny shown in Figure 2 applies the assumption of equal transition rates (assumption 1 above). For this analysis, the branch lengths of the consensus tree were scaled by 1/100 to make the units comparable to those of biological phylogenies; in theory this should not affect any calculations, but in practice we found that some models failed to converge because their parameter spaces are restricted to those sensible for biological rates measured over millions of years rather than the much faster cultural evolutionary rates.

These analyses use a maximum-likelihood framework, but Bayesian estimates of the root state are also possible. The 'Multistate' analysis used to describe transitions between states (see main text) also provides an estimate of the ancestral Pama-Nyungan grandparent system, in a model that allows transition rates to vary asymmetrically but is restricted to the four most common systems; for completeness, these results are also presented.

Binary Bayesian phylogenetic mixed models

These models fit logistic regressions with phylogenetic co-variance (under a Brownian motion model of evolution) as a random effect. We began each BBPMM with an initial burn-in on an arbitrary tree with 11,000,000 iterations to determine starting values, then sequentially ran on each tree 2,000,000 iterations, with a per-tree burn-in of 1,000,000 and

a sampling rate of 10,000, for a total posterior sample of 1,000 (10 for each tree). Priors for the fixed effects were set using the command 'gelman.prior' (Gelman et al., 2008) with a scale of $1 + \frac{\pi^2}{3}$; the prior for the phylogenetic random effect was set to be an inverse-Wishart distribution with $V = 1$ and $\nu = 0.02$. The residual variance was fixed at 1, which is standard for a logistic regression.

Non-phylogenetic models

Many studies of cross-cultural and cross-linguistic variation are not performed in a phylogenetic context, both historically and in present research. For our study to be comparable to such works, and to highlight the importance of phylogenetic comparative methods in the field of cultural evolution, we also tested our hypotheses in a non-phylogenetic framework, using Fisher's exact test.

When treating each ethnolinguistic society as an independent sample, we found no evidence of a correlation between grandparental term systems and linguistic exogamy ($p = 0.938$), nor between grandparental term systems and post-marital residence ($p = 1$). The observed link between grandparent-grandchild reciprocals and grandparental term systems, however, is statistically significant in a non-phylogenetic framework ($p < 0.001$), as are the links between grandchild and grandparental systems (specifically, that MG systems are more likely to also merge grandchildren terms by gender of the referent and less likely to merge by the identity of the parent, both $p < 0.001$).

Correlations between grandparental systems and community marriage organisation

In the main text, we present the correlation between the grandparental systems and community marriage organisation, with endogamy (few marriages outside the language group) and agamy merged into a single category and compared with exogamy (many marriages outside the language group). When instead agamy and exogamy are merged into a single group, and compared with endogamy, the results are similar.

Under this slightly different definition of community marriage organisation, there is also no relationship between this variable and whether the language distinguishes the female grandparents (pMCMC = 0.728), the male grandparents (pMCMC = 0.690), the parallel grandparents (pMCMC = 0.546), or the cross grandparents (pMCMC = 0.348).

When each language is considered an independent data point, there is no relationship between grandparental system and community marriage organisation (Fisher's exact test, $p = 0.634$); neither does a relationship exist in a phylogenetic framework (pMCMC = 0.974).

Data sources

All terms for grandparents and many of the terms for grandchildren and cross-cousins came from the online database of indigenous Australian languages, CHIRILA (http://chirila.yale.edu/) (Bowern, 2016) and the Austkin project (http://www.austkin.net/) (Dousset et al., 2010). We also consulted the following sources (with glottocodes in brackets, see Hammarström et al. 2018):

- Alyawarr (alya1239): Yallop (1977)
- Badimaya (badi1246): Dunn (1988)
- Bandjalang (band1339): Cunningham (1969); Geytenbeek and Geytenbeek (1971); Wafer et al. (2008)
- Biri (biri1256): Gargett (2011)
- Dharumbal (dhar1248): Terrill (2002)
- Diyari (dier1241): Austin (1981)
- Djapu (dhuw1249): Dixon and Blake (1979); Heath (1980)
- Dyirbal (mamu1253, dyir1250): Dixon (2015)
- Gamilaraay (gami1243): Giacon (2014), Wafer et al. (2008)
- Gumatj (dhuw1249, guma1253): Dixon and Blake (1979); Heath (1980)
- Gumbaynggir (kumb1268): Wafer et al. (2008)
- Gunya (guny1241): Wafer et al. (2008)
- Jaru (jaru1254): Tsunoda (1981)
- Karajarri (kara1476): Scheffler and Jorion (1981)
- KukuYalanji (kuku1273): Hershberger and Hershberger (1982); Patz (2002)
- MartuWangka (mart1256): Jones (2012)
- Muruwari (muru1266): Wafer et al. (2008)
- Ngadjumaya (ngad1258): Brandenstein (1980)
- Ngiyambaa (wang1291): Wafer et al. (2008)
- Paakantyi (darl1243): L. A. Hercus (1982); Wafer et al. (2008)
- PintupiLuritja (pint1250): Hansen (1978)
- Pitjantjatjara (pitj1243): Scheffler and Jorion (1981)
- Warlpiri (warl1254): Scheffler and Jorion (1981)
- Warumungu (waru1265): Luise Anna Hercus (1999)
- Warungu (waru1264): Scheffler and Jorion (1981)
- WikMungkan (wikm1247): Needham (1971)
- Wiradjuri (wira1262): Wafer et al. (2008)
- Yandruwandha (yand1253): Wafer et al. (2008)
- YortaYorta (yort1237): Wafer et al. (2008)

Table 1 in the main text defines the relevant terms with reference to the speaker. Some Australian languages also have trirelational kinship terms, where the propositus is defined in relation to another person. These terms affect which items are used for cognate reconstruction using linguistic comparative methods but do not affect the splits in systems discussed here.

Many of the community marriage organisation scores were obtained from Binford (2001), via the online cross-cultural database D-PLACE (https://d-place.org/) (Kirby et al., 2016). We also consulted the following sources:

- Adnyamathanha: Houseman (1997)
- Arabana: Hercus (1994); Nursey-Bray et al. (2013)
- Badimaya: Dunn (1988)
- Bandjalang: Harvey (2011)
- Batyala: Bell (2013)
- Bilinarra: Harvey and Garde (2016)
- CentralAnmatyerr: Young (1987)
- Dharawal: Toner (2015)
- Dharumbal: Morton (2017)
- Dhurga: Toner (2015)
- Djabugay: Henry (2000); McDonald and Lane (2000)
- Djinang: Waters (1984)
- Gupapuyngu: Munro and Nathan (2005); Vaughan (2018)
- GuuguYimidhirr: Haviland (1979)
- Jiwarli: Aikhenvald and Dixon (2001)
- KuguNganhcara: Baldi (2011)
- Kukatja: Denham (2013a); Dousset (2012)
- Kunjen: Ogilvie (1994); Sommer and Marsh (1969)
- KuukThaayorre: Gaby (2016); Harvey (2001)
- KuukuYau: Hill (2011, 2016, 2018)
- Mabuiag: Rivers (1900)
- MangalaMcK: Denham (2013b)
- Martuthunira: Dench (1995)
- Mudburra: Dorian (2004)
- Ngarinyman: Harvey and Garde (2016); Raynes (2000)
- Ngarluma: A. C. Dench (1995)
- Ngarrindjeri: Jenkin (1976)
- Nyamal: Read and Coppin (2014)
- Nyangumarta: Hargrave (1982)

- Panyjima: Dench (1987)
- Parnkala: Mathews (1900)
- PittaPitta: Conybeare (1898); Harvey (2011)
- Purduna: Goode et al. (2014); McConvell et al. (2018)
- Ritharrngu: Altman et al. (1999); Peterson and Myers (2016)
- Thanggati: Bowern (2010)
- Tharrgari: Dench (1995)
- Wangkangurru: Hercus (1994)
- Wardandi: Dousset et al. (2010); Gunn et al. (2011)
- Warlmanpa: Simpson (1985)
- Warnman: Tonkinson (1989, 2002)
- Warriyangga: Austin (2015)
- Warumungu: Simpson (2013)
- Yannhangu: Bowern and James (2005)
- YortaYorta: Bowe and Morey (1999)
- Yugambeh: Sharpe (1985, 1998)
- Yuwaalaraay: Austin (1993)

All post-marital residence data came from Moravec et al. (2018).

Supplemental references

Aikhenvald, A. Y., & Dixon, R. M. W. (2001). *Areal Diffusion and Genetic Inheritance: Problems in Comparative Linguistics* (A. Y. Aikhenvald & R. M. W. Dixon Eds. 1st edn edition ed.). Oxford ; New York: OUP Oxford.

Altman, J. C., Rowse, T., Morphy, F., & Reeves, J. (1999). *Land rights at risk? evaluations of the Reeves Report* (J. C. Altman, T. Rowse, F. Morphy, J. Reeves, & U. Australian National Eds.). Canberra: Centre for Aboriginal Economic Policy Research, Australian National University.

Austin, P. K. (1981). *A Grammar of Diyari, South Australia*. Cambridge: Cambridge University Press.

Austin, P. K. (1993). *A Reference Grammar of Gamilaraay, northern New South Wales*. Bundoora, Victoria: Department of Linguistics, La Trobe University.

Austin, P. K. (2015). *A Reference Grammar of the Mantharta Languages, Western Australia*: SOAS.

Baldi, P. (2011). *Linguistic Change and Reconstruction Methodology*: Walter de Gruyter.

Bell, J. (2013). The persistence of Aboriginal kinship and marriagerulesin Australia: Adapting traditional ways into modern practices. *The Journal of the European Association for Studies of Australia, 4*(1), 65-75.

Binford, L. R. (2001). *Constructing Frames of Reference: An Analytical Method for Archaeological Theory Building Using Ethnographic and Environmental Data Sets* (1st ed.). Berkeley: University of California Press.

Bowe, H. J., & Morey, S. (1999). *The Yorta Yorta (Bangerang) language of the Murray Goulburn: including Yabula Yabula*. Canberra: Pacific Linguistics, Research School of Pacific and Asian Studies, the Australian National University.

Bowern, C. (2010). Yan-nhaŋu language documentation and revitalisation. In J. R. Hobson (Ed.), *Re-awakening Languages*. Sydney: Sydney University Press.

Bowern, C. (2016). Chirila: contemporary and historical resources for the indigenous languages of Australia. *Language Documentation, 10*, 44.
Bowern, C., & James, B. (2005, 2015). *Yan-nhaŋu Revitalization: Aims and Accomplishments.* Paper presented at the Annual Meeting of the Chicago Linguistic Society.
Brandenstein, C. G. v. (1980). *Ngadjumaja: an Aboriginal language of south-east western Australia*: AMŒ.
Conybeare, F. C. (1898). Ethnological studies among the North-West-Central Queensland Aborigines by Walter E. Roth. *Folklore, 9*(3), 261-264.
Cunningham, M. C. (1969). *A description of the Yugumbir dialect of Bandjalang*: St. Lucia ; [Brisbane] : University of Queensland Press.
Dench, A. (1987). Kinship and collective activity in the Ngayarda languages of Australia. *Language in Society, 16*(3), 321-339. doi:10.1017/S0047404500012410
Dench, A. C. (1995). *Martuthunira: a language of the Pilbara Region of Western Australia* (Vol. 125). Canberra: ANU Press.
Denham, W. W. (2013a). Beyond fictions of closure in Australian Aboriginal kinship. *Mathematical Anthropology and Cultural Theory, 5*(1), 92.
Denham, W. W. (2013b). Response to comments on “Beyond fictions of closure in australian aboriginal kinship”. *5*(7), 13.
Dixon, R. M. W. (2015). *Edible Gender, Mother-in-Law Style, and Other Grammatical Wonders: Studies in Dyirbal, Yidiñ, and Warrgamay*. Oxford, New York: Oxford University Press.
Dixon, R. M. W., & Blake, B. J. (1979). *Handbook of Australian Languages: Volume 1: 001* (R. M. W. Dixon & B. J. Blake Eds.). Amsterdam: John Benjamins Publishing Company.
Dorian, N. C. (2004). Small languages and small language communities 45. *International Journal of the Sociology of Language, 168*, 141-156.
Dousset, L. (2012). ''Horizontal'' and ''vertical'' skewing: similar objectives, two solutions? In *Crow-Omaha: New Light on a Classic Problem of Kinship Analysis* (pp. 261-277): Amerind Studies in Anthropology.
Dousset, L., Hendery, R., Bowern, C., Koch, H., & McConvell, P. (2010). Developing a database for Australian Indigenous kinship terminology : the AustKin project. *Australian Aboriginal Studies, 1*, 42-56.
Dunn, L. (1988). Badimaya, a Western Australian language. *Papers in Australian Linguistics, 17*, 19-149.
Gaby, A. (2016). Hyponymy and the structure of Kuuk Thaayorre kinship*. In J.-C. Verstraete & D. Hafner (Eds.), *Culture and Language Use* (Vol. 18, pp. 159-178). Amsterdam: John Benjamins Publishing Company.
Gargett, A. D. (2011). *A Salvage Grammar of Malgana, the Language of Shark Bay, Western Australia*: Pacific Linguistics, School of Culture, History and Language, College of Asia and The Pacific, Australian National University.
Geytenbeek, B., & Geytenbeek, H. (1971). *Gidabal Grammar and Dictionary*: Australian Institute of Aboriginal Studies.
Giacon, J. (2014). *A grammar of Yuwaalaraay and Gamilaraay: a description of two New South Wales languages based on 160 years of records.* Available from ResearchGate
Goode, M. B., Wa, D., Huxtable, L., Road, S., & Wa, B. (2014). Report of an Aboriginal heritage survey of the North West Coastal Highway and materials pits SLK 690-766.03, in the Gnulli wc1997/028 and Budina wc2004/005 NTC: Gascoyne Region, Western Australia. 81.
Gunn, R. G., Dortch, J., Ogleby, C., & Thorn, A. (2011). The petroglyphs of the Kybra Aboriginal Site, South-Western Western Australia. *Journal of the Royal Society of Western Australia, 94*, 26.
Hammarström, H., Bank, S., Forkel, R., & Haspelmath, M. (2018). Glottolog 3.2. Retrieved from http://glottolog.org. from Max Planck Institute for the Science of Human History http://glottolog.org
Hansen, L. E. (1978). *The Core of Pintupi grammar*: Institute for Aboriginal Development.

Hargrave, S. (1982). *Language and Culture* (S. Hargrave Ed. Vol. 8). Darwin: Summer Institute of Linguistics.
Harvey, M. (2001). Oenpelli Kunwinjku Kinship Terminologies and Marriage Practices. *Oceania*.
Harvey, M. (2011). Lexical change in pre-colonial Australia. *Diachronica, 28*(3), 345-381. doi:10.1075/dia.28.3.03har
Harvey, M., & Garde, M. (2016). Matries and bubsections: bodies and social personae in Northern Australia. *Anthropological Linguistics, 57*(3), 229-274. doi:10.1353/anl.2016.0006
Haviland, J. B. (1979). Guugu Yimidhirr brother-in-law language. *Language in Society, 8*(2-3), 365-393. doi:10.1017/S0047404500007600
Heath, J. (1980). *Dhuwal (Arnhem Land) texts on kinship and other subjects, with grammatical sketch and dictionary*: University of Sydney.
Henry, R. (2000). Dancing into Being: The Tjapukai Aboriginal Cultural Park and the Laura Dance Festival. *The Australian Journal of Anthropology, 11*(3), 322-332.
Hercus, L. A. (1982). *The Bagandji Language*. Canberra, A.C.T., Australia: Dept. of Linguistics, Research School of Pacific Studies, Australian National University.
Hercus, L. A. (1994). *A Grammar of the Arabana-Wangkangurru Language, Lake Eyre Basin, South Australia*: Pacific Linguistics.
Hercus, L. A. (1999). *A Grammar of the Wirangu Language from the West Coast of South Australia*: Pacific Linguistics.
Hershberger, H. D., & Hershberger, R. (1982). *Kuku-Yalanji dictionary*: Darwin : Summer Institute of Linguistics, Australian Aborigines Branch.
Hill, C. (2011). Named and Unnamed Spaces: Color, Kin, and the Environment in Umpila. *The Senses and Society, 6*(1), 57-67. doi:10.2752/174589311X12893982233759
Hill, C. (2016). Expression of the interpersonal connection between narrators and characters in Umpila and Kuuku Ya'u storytelling. *Narrative Inquiry, 26*(2), 257-285. doi:10.1075/ni.26.2.04hil
Hill, C. (2018). *Person reference and interaction in Umpila/Kuuku Ya'u narrative.* Radboud Universiteit Nijmegen,
Houseman, M. (1997). Marriage networks among Australian Aboriginal populations. *Australian Aboriginal Studies, 2*, 1-23.
Jenkin, G. (1976). *The Aborigines' Friends' Association and the Ngarrindjeri People.* University of Adelaid,
Jones, B. J. (2012). *A grammar of Wangkajunga : a language of the Great Sandy Desert of north Western Australia*. Canberra: Pacific Linguistics.
Kirby, K. R., Gray, R. D., Greenhill, S. J., Jordan, F. M., Gomes-Ng, S., Bibiko, H.-J., . . . Gavin, M. C. (2016). D-PLACE: a global database of cultural, linguistic and environmental diversity. *PLOS ONE, 11*(7), e0158391. doi:10.1371/journal.pone.0158391
Mathews, R. H. (1900). Divisions of the South Australian Aborigines. *Proceedings of the American Philosophical Society, 39*(161), 78-91.
McConvell, P., Kelly, P., & Lacrampe, S. (2018). *Skin, Kin and Clan*: ANU Press.
McDonald, G., & Lane, M. (2000). *Securing the Wet Tropics - a Retrospective on Managing Australia's Tropical Rainforests* (1 edition ed.). Leichhardt, N.S.W: Federation Press.
Moravec, J. C., Atkinson, Q., Bowern, C., Greenhill, S. J., Jordan, F. M., Ross, R. M., . . . Cox, M. P. (2018). Post-marital residence patterns show lineage-specific evolution. *Evolution and Human Behavior, 39*(6), 594-601. doi:10.1016/j.evolhumbehav.2018.06.002
Morton, J. (2017). 'Mother's Blood, Father's Land': native title and comparative land tenure modelling for claims in 'settled' Australia. *Oceania, 87*(1), 58-77. doi:10.1002/ocea.5150
Munro, R., & Nathan, D. L. (2005, 2005). *Towards portability and interoperability for linguistic annotation and language-specific ontologies*.
Needham, R. (1971). *Rethinking kinship and marriage*: Tavistock Publications.

Nursey-Bray, M., Fergie, D., Arbon, V., Rigney, L.-I., Palmer, R., Tibby, J., . . . Hackworth, L. (2013). *Community based adaptation to climate change: the Arabana, South Australia*: National Climate Change Adaptation Research Facility.

Ogilvie, S. (1994). *The Morrobalama (Umbuygamu) language of Cape York.* The Australian National University, Canberra, A.C.T.

Patz, E. (2002). *A Grammar of the Kuku Yalanji Language of North Queensland*: Pacific linguistics, Research School of Pacific and Asian Studies, the Australian National University.

Peterson, N., & Myers, F. (2016). *Experiments in self-determination* (N. Peterson & F. Myers Eds.). Canberra: ANU Press.

Raynes, C. M. (2000). *The moral sub-text of Aboriginal oral history frmo the Western Victorial River Region, Northern Territory.* Northern Territory University,

Read, J., & Coppin, P. (2014). Kangkushot: The Life of Nyamal Lawman Peter Coppin. In *Murrkanya* (pp. 30-36). Canberra: Aboriginal Studies Press.

Rivers, W. H. R. (1900). A Genealogical Method of Collecting Social and Vital Statistics. *The Journal of the Anthropological Institute of Great Britain and Ireland, 30*, 74. doi:10.2307/2842619

Scheffler, H. W., & Jorion, P. (1981). Australian Kin Classification. *Man, 16*(1), 139-142.

Sharpe, M. C. (1985). Bundjalung Settlement and Migration. *Aboriginal History, 9*(1), 101-124.

Sharpe, M. C. (1998). *Dictionary of Yugambeh*. Canberra: ANU Press.

Simpson, J. (1985). How Warumungu people express new concepts. *Language in Central Australia, 4*, 12-25.

Simpson, J. (2013). Warumungu kinship system over time. In P. McConvell, I. Keen, & R. Hendery (Eds.), *Kinship Systems: Change and reconstruction* (pp. 239-254). Utah: University of Utah Press.

Sommer, B. A., & Marsh, J. (1969). Vernacular and English: Language Comprehension by Some North Queensland Aborigines. *Anthropological Linguistics, 11*(2), 48-57.

Terrill, A. (2002). *Dharumbal: the language of Rockhampton, Australia*: Pacific Linguistics, Research School of Pacific and Asian Studies, Australian National University.

Toner, P. G. (2015). *Strings of Connectedness*: ANU Press.

Tonkinson, R. (1989). Local organisation and land tenure in the Karlamilyi (Rudall River) region. In *The Significance of the Karlamilyi Region to the Martujarra of the Western Desert*. Perth: WA Department of Conservation and Land Management.

Tonkinson, R. (2002). *The Mardu Aborigines: Living the Dream in Australia's Desert* (2nd ed.). Mason, Ohio: Wadsworth Pub Co.

Tsunoda, T. (1981). *The Djaru language of Kimberley, Western Australia*. Canberra, A.C.T: Dept. of Linguistics, Research School of Pacific Studies, Australian National University.

Vaughan, J. (2018). “We talk in saltwater words”: Dimensionalisation of dialectal variation in multilingual Arnhem Land. *Language & Communication, 62*, 119-132. doi:10.1016/j.langcom.2017.10.002

Wafer, J., Lissarrague, A., & Harkins, J. (2008). *A handbook of Aboriginal languages of New South Wales and the Australian Capital Territory*. Nambucca Heads, N.S.W.: Muurrbay Aboriginal Language and Culture Co-operative.

Waters, B. (1984). *Djinang and Djinba : a grammatical and historical perspective*. Canberra: Australian National University.

Yallop, C. (1977). *Alyawarra, an Aboriginal language of central Australia*: Australian Institute of Aboriginal Studies.

Young, E. (1987). Resettlement and caring for the country: the Anmatyerre experience. *Aboriginal History, 11*(2), 156-170.
